# Medicinal Plants Used for Treatment of Diarrhoeal Related Diseases in Ethiopia

**DOI:** 10.1155/2018/4630371

**Published:** 2018-03-18

**Authors:** Bizuneh Woldeab, Reta Regassa, Tibebu Alemu, Moa Megersa

**Affiliations:** ^1^Department of Environmental Health Science and Technology, Jimma University, P.O. Box 378, Jimma, Ethiopia; ^2^Department of Biology, Hawassa College of Teacher Education, P.O. Box 115, Hawassa, Ethiopia

## Abstract

This paper presents a review of relevant antidiarrhoeal medicinal plants based on the fundamental knowledge accumulated by indigenous people of Ethiopia. The review includes an inventory carried out on the phytochemical and pharmacological analysis of plant species used in the treatments of diarrhoeal diseases. This study is based on a review of the literature published in scientific journals, books, theses, proceedings, and reports. A total of 132 medicinal plants used by local people of Ethiopia are reported in the reviewed literature. Herbs (43.6%) were the primary source of medicinal plants, followed by trees (27%). Some findings include the predominance of leaf material used (78%), as well as the frequent use of crushing of the plant parts (38%) as a mode of preparation. This study demonstrates the importance of traditional medicines in the treatment of basic human ailments such as diarrhoeal diseases in Ethiopia. Baseline information gaps were observed in different regions of Ethiopia. Thus, documentation of the knowledge held by other regions of Ethiopia that have so far received less attention and urban ethnobotany is recommended for future ethnobotanical studies. In addition, phytochemical studies are recommended mainly on frequently utilized medicinal plants for treatment of diarrhoeal diseases which can serve as a basis for future investigation of modern drug development. Although societies in Ethiopia have long used medicinal plants for diarrhoeal diseases treatment, it is also a good practice to perform toxicological tests.

## 1. Introduction

Diarrhoea is a leading killer of children, accounting for nine percent of all deaths among children under age of five worldwide in 2015 [1]. Sub-Saharan Africa and southern Asia were the regions with the highest child death rates due to diarrhoea in 2015 [2]. In these regions, children under five experience 3.2 to 12 episodes of diarrhoea every year [2]. It is also reported that millions of people were at risk of diarrhoea in Ethiopia, where acute watery diarrhoea broke out in crowded and unsanitary conditions of urban and rural areas in 2009 [3]. To fight this problem, the World Health Organization (WHO) has initiated a diarrhoea disease control program to study traditional medicine practices and prevention approaches [4]. This may have valuable advantages in reducing mortality rate in developing countries due to diarrhoea.

In Ethiopia, plants have been used as a source of medicine from time immemorial to treat different ailments and traditional medicine is an integral part of Ethiopian culture [5]. Traditional remedies are the most important and sometimes the only source of therapeutics for nearly 80% of the Ethiopian population and 95% of the preparations are of plant origin [6]. The widespread use of traditional medicine in communities of Ethiopia could be attributed to cultural acceptability, efficacy, physical accessibility, and economic affordability as compared to modern diarrhoeal medicine [6]. Study on traditional remedies in Ethiopia demonstrates that diarrhoea is one of the most prominent diseases treated by traditional medicines [7–9].

Although there are a range of medicinal plants with antidiarrhoeal properties that have been widely used by local communities of Ethiopia, the effectiveness of many of these antidiarrhoeal traditional medicines has not been scientifically evaluated [10]. Recently, a few of these medicinal plants have attracted considerable attention and studies being conducted to scientifically evaluate their antidiarrhoeal activities. For instance, the antidiarrhoeal activities of* Calpurnia aurea *[11],* Cordia africana *[12],* Indigofera spicata* [13],* Lepidium sativum *[14],* Stereospermum kunthianum *[15],* Vernonia amygdalina *[16], and* Zehneria scabra *[17] have been investigated on experimental animals.

It has been reported in various studies [9, 18, 19] that the knowledge on medicinal plants is getting lost due to the lack of interest by the younger generation. Since the knowledge of traditional medicine is transferred orally from generation to generation, the basic information about part of the plants used, drug preparation methods, the diseases treated, and others may be lost and discarded amidst the knowledge transfer process. Therefore, documentation of these medicinal plants is important in order to identify candidate species for the invention of therapeutic drugs. Thus, this review is initiated to document traditional uses of medicinal plants to treat diarrhoeal diseases in Ethiopia, to provide comprehensive documentation, to identify research gaps, and to suggest perspectives for future research.

## 2. Methods

The traditional uses of medicinal plants used to treat diarrhoeal diseases in Ethiopia were collected from available literature published in scientific journals, books, theses, proceedings, and reports. Literature was searched in different electronic databases (Web of Science, MEDLINE, Science Direct, and Google Scholar) and accessed between April 2016 and September 2017 using specific search terms such as “medicinal plants”, “traditional medicines”, and “Ethiopia or Indigenous people”. After identifying potential literatures, we searched if there is a report of medicinal plants used for treatment of diarrhoea in the region where the study was carried out. Data collected from the literature includes mode of preparation and administration of the species, plant parts used, additives, condition, and dosages used. Moreover, literature search was also done to document the biological and pharmacological activities of the mostly used plant species for treatment of diarrhoea.

**Figure 1:**
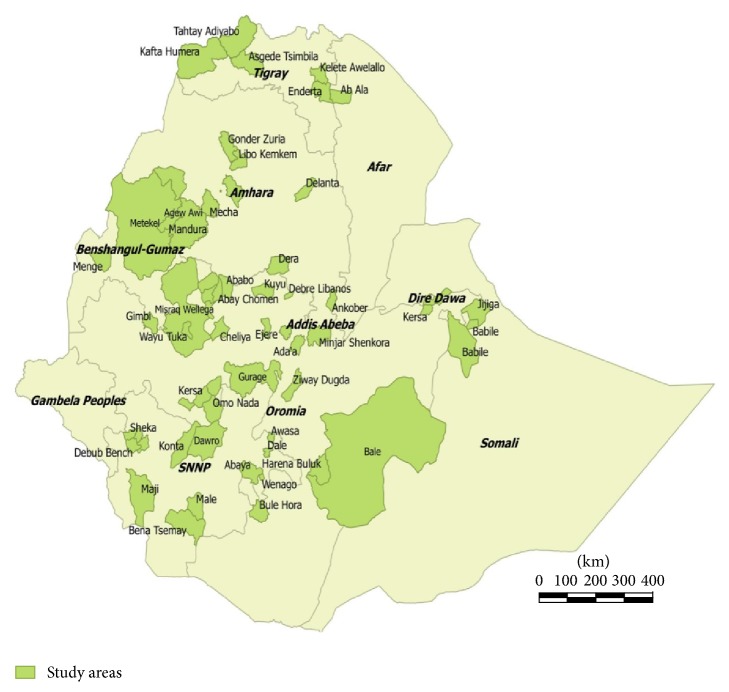
Location of study sites in Ethiopia.

We reviewed a total of 58 publications that provided information about the use of medicinal plant species to treat diarrhoeal diseases in Ethiopia (Figure 1). There are papers reported about the use of medicinal plant species from all regions of Ethiopia published between 1965 and 2017.

## 3. Medicinal Plants Diversity

This study recorded one hundred thirty-two plant species as useful in traditionally managing diarrhoeal diseases in Ethiopia (Table 1). These medicinal plants were distributed among 52 families and 113 genera. Most of medicinal plants, 74 (56%), used in Ethiopia for diarrhoeal treatment are from 29 families (Table 2). Plant families with the highest number of medicinal plants in Ethiopia used for diarrhoeal diseases treatment were Fabaceae and Lamiaceae (12 species each), followed by Asteraceae (10 species), Euphorbiaceae (7 species), Verbenaceae, Solanaceae Malvaceae, Ranunculaceae, Poaceae, and Cucurbitaceae (4 species each). Fabaceae, Lamiaceae, and Asteraceae families have the highest number of species used as herbal medicines. The rest of the families were represented by one, two, or three species each (Table 2). The genus with the highest number of species was Ajuga with three species.

### 3.1. Growth Forms and Parts Used

**Figure 2:**
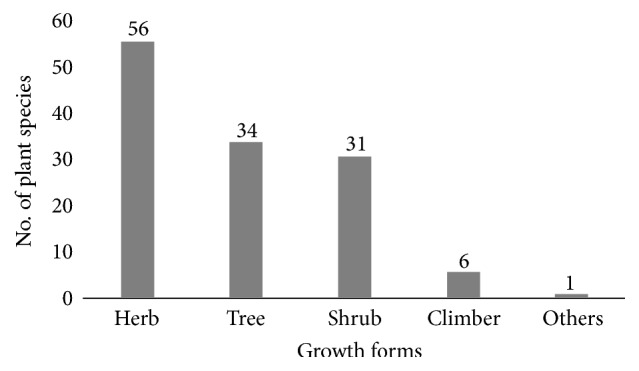
Growth forms (habits) of medicinal plants.

**Figure 3:**
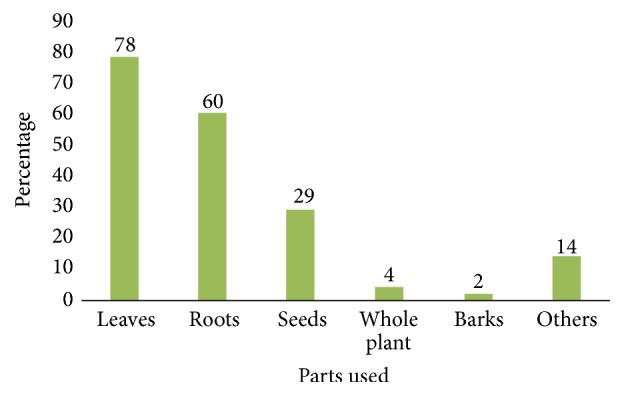
Plant parts used for the treatment of diarrhoeal diseases in Ethiopia.

The result of growth form analysis of medicinal plants shows that herbs constitute the highest proportion being represented by 56 (43.6%) species, while there were 34 (27%) tree species, 31 (20.6%) shrubs, and 6 (2%) climbers (Figure 2). Almost all plant parts, the roots, rhizomes, stem, bark, leaves, flowers, fruits, young shoots, and whole plants, were used to prepare different remedies (Table 1). The most frequently used plant parts were leaves (78%), followed by roots (60%), and seeds (29%), and others constitute 20% (Figure 3).

### 3.2. Methods of Preparation of Traditional Medicine

Most of the medicinal plant preparation involved the use of single plant species or a single plant part (76%), whereas the preparation via mixing different plants or plant parts (24%) is rarely encountered in Ethiopia. In Ethiopia, the most common methods of preparation of traditional medicine from plant materials are crushing (38%), followed by decoction (21%) and others (Table 3).

### 3.3. Condition, Additives, and Dosages of Medicinal Plants

Most of the remedies (58%) used in Ethiopia for treatment of diarrhoeal diseases are prepared from fresh parts of medicinal plants followed by drying form 27% and 15% prepared either from dry or fresh plant parts. Various additives are used during administration of traditional medicine. Additives like honey (48%), salt (13%), sugar (13%), local beer (3%), milk (16%), and butter (6.5%) are used with traditional medicine to increase the flavor, taste, and general suitability of orally administered remedies.

People living in rural areas of Ethiopia use various ways of measuring dosage which are generally categorized under three major classes. One is dosage used for those medicinal plants which are expected to be highly toxic. For such medicines, the measurement can be undertaken by little finger index and very little amount of the prepared medicine taken by half a coffee cup. For example, medicines prepared from* Cordia africana*,* Calpurnia aurea*,* Verbena officinalis, *and* Brucea antidysenterica *are toxic if overdosed. The second is the dosage used for medicinal plants which can have little effect on human health if overdosed. The dosage is measured by hand palm and taken by bottle or locally made material from* Lagenaria siceraria*. Examples of species include* Delphinium dasycaulon* and* Entada abyssinica*. In the third case, there are medicinal plants that do not have any observable side effect. Medicines prepared from* Zingiber officinale*,* Coffea arabica*,* Syzygium guineense, Guizotia abyssinica, *and* Citrus limon* can be taken according to the existing traditional practice of communities and/or personal preference.

### 3.4. Frequently Reported Medicinal Plants

Among the most commonly used plants were* Calpurnia aurea*,* Coffea arabica*,* Cordia africana*,* Rumex nepalensis*,* Zehneria scabra*,* Verbena officinalis*,* Verbascum sinaiticum*,* Amaranthus caudatus*,* Vernonia amygdalina*,* Stereospermum kunthianum*,* Lepidium sativum*,* Indigofera spicata*,* Syzygium guineense*,* Leucas deflexa*,* Citrus limon,* and* Brucea antidysenterica* are some to mention.

### 3.5. Phytochemical and Pharmacological Studies of Mostly Reported Medicinal Plants

The most frequent approach to species selection for phytochemical, pharmacological, or antimicrobial analysis is by reviewing the ethnobotanical literature [72]. In this approach, various modern drugs were extracted from traditional medicinal plants using plant materials [73]. Plant-based traditional medicine is still playing a vital role in the development and advancement of modern studies by serving as a starting point for the development of novelties in drug discovery [74]. The presence of active ingredients such as flavonoids, terpenoids, steroids, phenolic compounds, and alkaloids in herbal medicines has been reported to have antimicrobial activity and directly linked to their ability to prevent or treat ailments such as diarrhoeal diseases [75, 76]. Phytochemical analysis of plant preparations and the identification of active components therein is also helpful to explain the mechanism of antidiarrhoeal activity [10]. For instance, polyphenols and tannins provide strength to intestinal mucosa, decrease intestinal secretion, and promote balance in water transportation across the mucosal cells [77]. Flavonoids and saponins inhibit the release of prostaglandins, autacoids, and contractions caused by spasmogens as well as motility and hydroelectrolytic secretions [78]. The phytochemical analysis of mostly cited medicinal plants used for treatment of diarrhoeal diseases in Ethiopia will be discussed below. Few of these plants have been validated as treatments for diarrhoea based on their ability to prevent diarrhoeal symptoms induced in experimental animals.

#### 3.5.1. *Calpurnia aurea* (Ait.) Benth.

**Figure 4:**
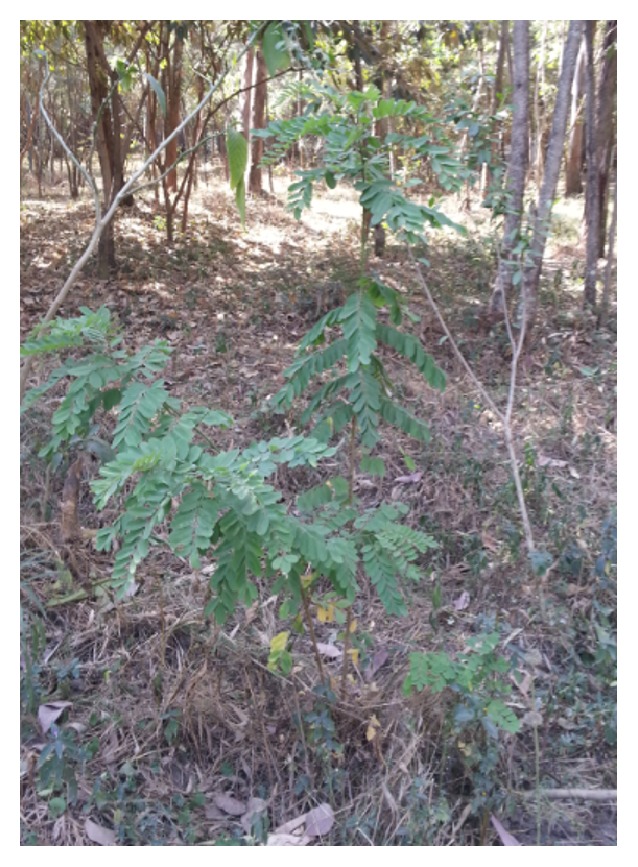
*C. aurea *(Courtesy: [Regassa, Hawassa, Ethiopia, 2016]).

It is shrub or small tree commonly growing up to 5 m in bushlands and occasionally up to 10 m in forests of Ethiopia. The plant grows in different habitats, such as grazing lands, roadsides, and homegardens [9, 18].* C. aurea* is widespread throughout the highlands from 1400 to 2500 m where rainfall is from 1000 to 2000 mm per year [79]. Trees are found in flower throughout the year but most abundantly after the small rains in May [79]. The leaves of* C. aurea* (Figure 4) are used traditionally in many parts of Ethiopia to treat diarrhoeal diseases. The use of* C. aurea* for diarrhoeal diseases by local people of Ethiopia was reported 14 times by researchers. Methanol extracts of leaf revealed the presence of alkaloids, tannins, flavonoids, and saponins [11]. Plants species possessing alkaloids, tannins, flavonoids, steroids, and terpenoids exhibit antidiarrhoeal activity [80]. Alkaloids demonstrated inhibitory effect on Nitric oxide synthesis [81]. Tannins and flavonoids have been reported to have antidiarrhoeal activity through inhibition of intestinal motility [82] and hydroelectrolyte secretion [83]. The leaf extracts of* C. aurea* have exhibited dose (100–400 mg/kg) dependent inhibition of castor oil induced diarrhoea on experimental mice like loperamide [11]. More interestingly, studies also confirmed that extracts from the species have broad spectrum antibacterial activity [84]. Being broad spectrum is advantageous in reducing diarrhoea as there are various causative agents of the disease.

#### 3.5.2. *Zehneria scabra* (Linn. f.) Sond.

**Figure 5:**
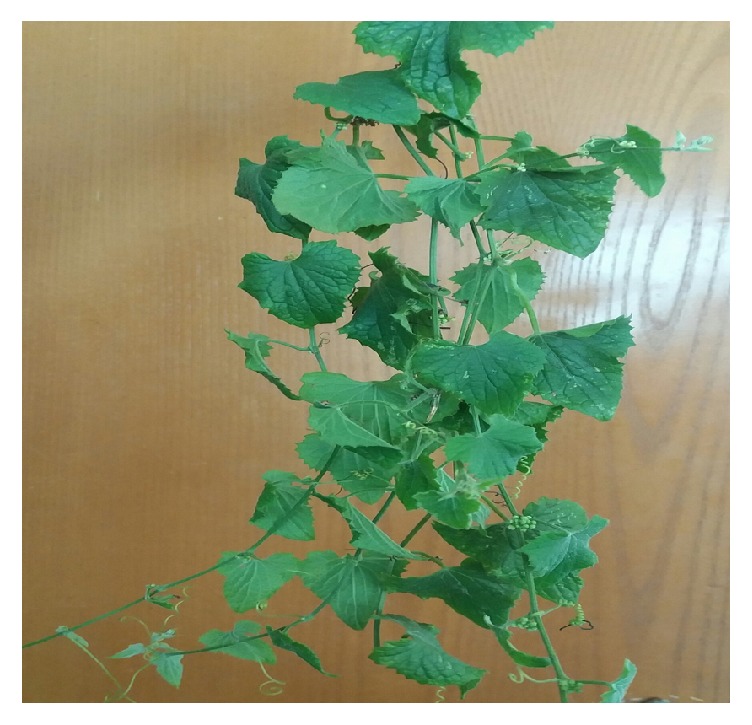
*Z. scabra *(Courtesy: [Woldeab, Jimma, Ethiopia, 2017]).

*Z. scabra* is one of the commonly used medicinal plants in Ethiopian traditional medicine practices (Figure 5). Local people in Ethiopia use the leaves of the plant to get relief from diarrhoea [43]. The plant is also used for the treatment of wound, alopecia, and eczema in folk medicines [85]. The phytochemical study conducted by [17] showed the presence of tannins, saponins, anthraquinones, O-anthraquinones, and phenols in the crude extract of leaves of* Z. scabra*. The phytochemical study of root extracts of* Z. scabra* by [86] also revealed the presence of Gypenoside. China pharmaceutical company manufactures Gypenoside tablets; it is used as a supplemental medicine for treating various diseases. The methanolic extracts of* Z. scabra* showed antidiarrhoeal activity in mice [17]. Studies also report that ethanol, methanol, ethyl acetate, and aqueous extracts of* Z. scabra* show maximum zone of inhibition for standard strains of* Staphylococcus aureus *[86].

#### 3.5.3. *Stereospermum kunthianum* Cham.

It is a small tree occurring at medium to low altitudes, frequently on rocky outcrops and hillsides. It also occurs in open woodlands and at margins of evergreen forests [87] and is found from 700 to 2000 m in areas with an annual rainfall range between 700 and 1800 mm per year [79]. Flowering most abundantly after the small rains in April and May, some trees can be found with flowers any time from September up to May [79].* S. kunthianum *is only found in the wild and increasingly becoming difficult to harvest due to high scarcity [54]. The root and bark of the plant* S. kunthianum* are used in diarrhoea treatment in Ethiopia [31, 35]. Preliminary phytochemical screening of the leaves extract of* S. kunthianum* reveals the presence of sterols/triterpenes, saponins, tannins, coumarins, and free carboxylic group [88]. The methanolic extracts from this plant species exhibited antidiarrhoeal activity on mice. The effect is comparable to loperamide, which is presently one of the most widely used antidiarrhoeal drugs [88]. The antidiarrhoeal effect of a flavonoid isolated from the stem bark aqueous extract of* S. kunthianum *is investigated using rodent models of castor oil-induced gastrointestinal motility and castor oil-induced diarrhoea [15]. The result indicates that pretreatment with dimethoxyflavone causes a delay in the onset of diarrhoea, reduction in the frequency, and weight of wet stools [15]. The result from this study suggests that the antidiarrhoeal activity of dimethoxyflavone from* S. kunthianum *castor oil-induced diarrhoea in mice is probably due to antielectrolyte permeability action [15]. Toxicity studies of the aqueous extract of* S. kunthianum* stem bark show that the extract has a wide safety margin [89].

#### 3.5.4. *Lepidium sativum* L.

**Figure 6:**
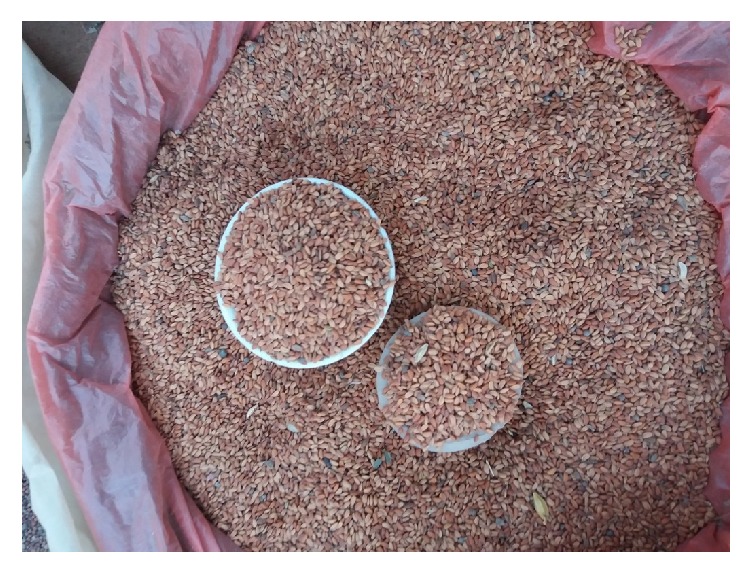
*L. sativum *seeds (Courtesy: [Regassa, Hawassa, Ethiopia, 2016]).

It occurs in all regions of Ethiopia at altitudes between 750 and 2900 m [90].* L. sativum *is not widely cultivated; instead it grows with other crops, particularly* Eragrostis tef* (Zucc.) Trotter and is available in all local markets [79]. In different regions of Ethiopia,* L. sativum* seeds (Figure 6) are used commonly to treat abdominal discomfort, such as dysentery and diarrhoea [9, 42]. A study conducted by [91] indicated antidiarrhoeal effect of alcoholic and aqueous extract of* L. sativum *seeds in three animal models (castor oil induced diarrhoea in rats, prostaglandin induced enteropooling in rats, and charcoal meal test in mice) of diarrhoea. Furthermore, the study found aqueous extract to be more potent than alcoholic extract.* L. sativum* has been used widely in different parts of the world for its wide therapeutic application [14]. The plant is well studied phytochemically with various active components, such as alkaloids, riboflavin, and ascorbic, linoleic, oleic, palmitic, and stearic acids [92]. Moreover, cucurbitacin has also been identified as plant constituent [93]. The plant has been shown to exhibit antidiarrhoeal and antispasmodic effects in rats, mediated through dual blockade of muscarinic receptors and Ca^++^ channels [14]. The findings of [94] suggest that the aqueous methanol extracts of seeds of* L. sativum* exhibit antidiarrhoeal activity through activation of K^+^ channels and inhibition of PDE enzyme in rabbit intestine.

#### 3.5.5. *Vernonia amygdalina* Del.

**Figure 7:**
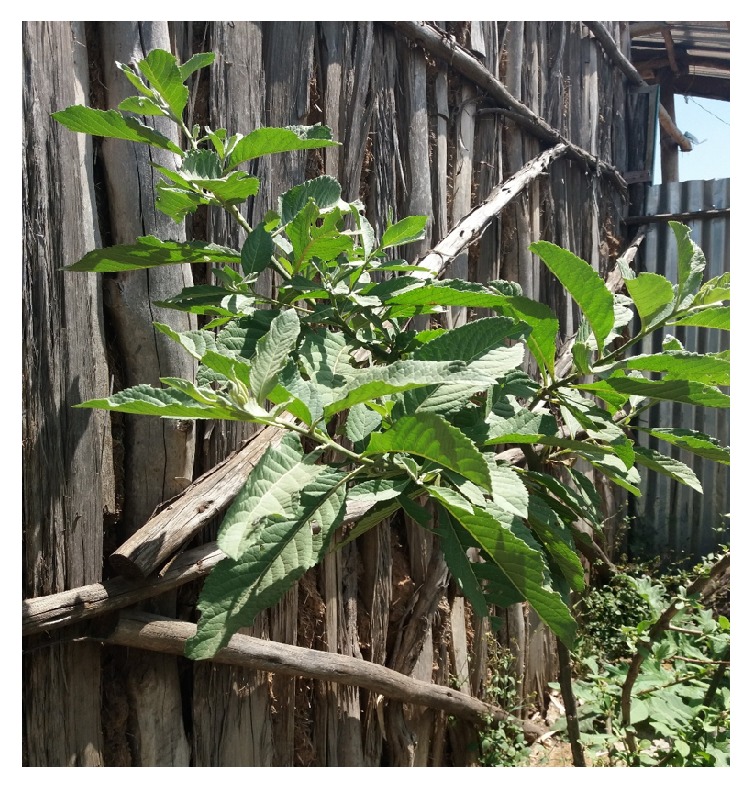
*V. amygdalina *(Courtesy: [Megersa, Gute, Ethiopia, 2013]).

It is found in a wide range of bushlands, woodlands, and forest habitats between 500 and 2800 m; it is also often found around home. Its rainfall range is from 750 to 2000 mm per year [95]. Flowering trees can be found from December to May, but the main flowering period is from January to February [79]. Some observations showed that the plant is widely available and frequently growing in homegardens [7, 9, 17, 33, 69]. The leaves of* V. amygdalina *(Figure 7) are used in Ethiopian folk medicine to treat malaria [7, 9] and gastrointestinal disorders [28, 42, 58]. The leaf extract is reported to reduce the symptoms of castor oil-induced diarrhoea in rats and reduce gastrointestinal transit of a charcoal meal in mice [10]. Methanolic extract of* V. amygdalina *shows that the 400 mg/kg body weight administered orally to the experimental rats was effective in the reduction of the faecal spots [16]. A qualitative phytochemical analysis revealed the presence of some important active components such as saponins and alkaloids [16, 96], tannins [16], flavonoids [97], and glycosides [16]. Some of these components might be responsible for the antidiarrhoeal properties [96]. Therefore, this might indicate that the antidiarrhoeal activities are the most likely combined effect or a single effect [16]. Moreover, the other biomolecules may be neither synergistic nor antagonistic.

#### 3.5.6. *Cordia africana* Lam.

It is found in moist evergreen highlands and riverine forests of the northwest and southwest highlands and very common in western Ethiopia between 550 and 2600 m where the rainfall range is 700 to 2000 mm per year [79]. Some trees can be found in flower at any time of the year, but the main flowering period is from October to March [87].* C. africana* is the most preferred plant by local people of Ethiopia for various uses, such as construction, timber production, charcoal, and medicine [9, 54, 55]. Due to its multipurpose uses,* C. africana* is becoming threatened, which is evidently shown by its scarce distribution in different areas of Ethiopia [9, 54, 98].* C. africana* is one of indigenous tree species that have currently been given conservation priority by using in situ and ex situ conservation in the country [99].

**Figure 8:**
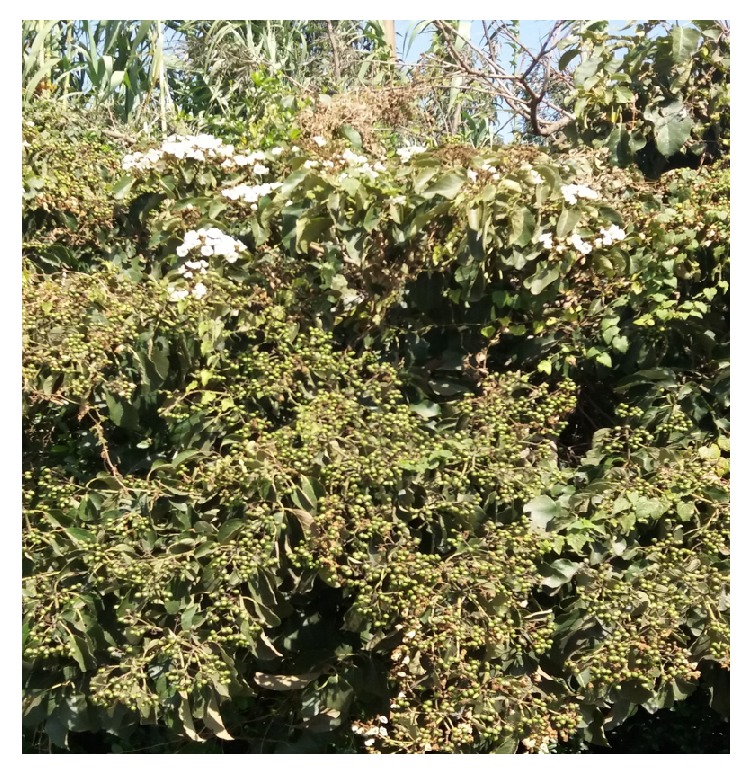
*C. africana *(Courtesy: [Megersa, Gute, Ethiopia, 2013]).

In an ethnobotanical study,* C. africana *(Figure 8) is reported to be used in the management of diarrhoea in Shinasha, by Agew-Awi, Oromo, Tigray, and Amhara people in Ethiopia [28, 31, 37, 55]. The phytochemical analysis of methanolic root bark extracts of* C. africana *revealed that the plant material contains phenols, saponins, tannins, terpenoids, and flavonoids [100]. The mechanism of action of saponin for antidiarrhoeal effect is through the inhibition of histamine; terpenoids inhibit release of autacoids and prostaglandins; phenol makes intestinal mucosa more resistant [12]. There are considerable reports on* C. africana* studies showing the antidiarrhoeal effects on experimental animals [12, 101] For instance, the study conducted by [101] indicated the significant reduction of castor oil induced diarrhoea compared to loperamide. The study also revealed that the extract significantly reduced intestinal transit like that of atropine.

#### 3.5.7. *Indigofera spicata* Forssk.

*I. spicata* is traditionally used for the treatment of meningitis [18], diarrhoea [20, 23], stomach-ache [35], diabetes [9], and other health problems in Ethiopia. Phytochemical studies conducted on this plant species indicated the presence of alkaloids, flavonoids tannins, and steroids in this plant [13, 102]. Steroids reported to enhance the absorption of hydroelectrolytes across the intestinal lumen [75]. In the castor oil induced diarrhoea in mice, the root extract of* I. spicata* inhibits frequency of defecation and fluid content of stool more likely in a dose dependent manner [13]. Moreover, the study found 53.51% inhibition of frequency of defecation which was equivalent to 51.02% of the positive control (atropine). The demonstrated biological antidiarrhoeal activity of crude root extracts of* I. spicata* could be due to the presence of these constituents [13]. Besides its use as antidiarrhoeal, usage of this plant raises the safety concern because the plant has showed effect on liver and cause abortion [103] and teratogenic effects in animals [104].

### 3.6. Prospects and Viewpoints

This review showed that local people in Ethiopia rely on traditional medicines to treat diarrhoeal diseases and are knowledgeable about the applications of medicinal plants. Many people in Ethiopia are still dependent on medicinal plants, at least for the treatment of basic human ailments. Diarrhoea is a major concern in Ethiopia and it is one of the basic human ailments frequently treated by medicinal plants in many regions of Ethiopia [8, 9, 46]. The finding correlates strongly with observations made by [105] that diarrhoea is a major concern in Mozambique [106], in South Africa, and [76] in south-central Zimbabwe. Reports of similar medicinal applications of the documented plants in Ethiopia and the rest of the world indicate that these species are valuable sources of ethnomedicines.

The current review addresses the existence of traditional indigenous knowledge. This considerable indigenous knowledge, from the earliest times, is linked with the use of traditional medicine in many countries including Ethiopia [107]. Indigenous knowledge refers to the knowledge, innovations, and practices of indigenous and local communities around the world [108]. Thus, there is a growing appreciation of the value of traditional knowledge and its potential benefits to modern industry [108]. Many widely used products, such as plant-based medicines for human and livestock health, have been developed using indigenous traditional knowledge. It is, therefore, necessary to preserve this indigenous knowledge on traditional medicines by proper documentation, identification of plant species used, herbal preparation, and dosage. This review will assist future studies on the selection of herbal plants to evaluate for phytochemical safety and pharmaceutical efficacy.

Almost all ethnobotanical studies in Ethiopia have so far focused on medicinal plants used by rural communities. Urban ethnobotany is minimal or lacking as almost all studies gave with consideration to ethnobotanical studies in rural areas. Applied to the context of immigration, urban ethnobotany will offer the opportunity to evaluate ethnobotanical data within a larger, transnational framework by comparing plant knowledge and use by the same cultural group in its original and new environment [109]. The paradigm shift is also needed from ethnobotanical studies to phytochemical and pharmacological studies. For instance,* C. arabica* was reported by eight authors,* R. nepalensis *by six authors,* V. officinalis *by five authors, and* V. sinaiticum* by four authors used to treat diarrhoeal diseases in Ethiopia, but no study validated their antidiarrhoeal property by investigating the biological activity of the extracts. Along with phytochemical and pharmacological studies, ethnobotanical studies should also continue, particularly in areas that have received less attention so far. For example, studies should be conducted in the Afar, Benishangul Gumuz, Gambela, Somali, and Tigray regions, like that of Amhara, Oromia, and SNNPR regions (Figure 1).

Almost all ethnobotanical studies conducted in Ethiopia have an objective of documentation of medicinal plants used to treat various diseases including diarrhoea. Early ethnobotanical studies conducted in Ethiopia simply focused on listing the name of medicinal plant species with diseases treated [45, 58, 68] without providing any detailed information on the medicinal plants in the region. Another concern of ethnobotanical studies conducted in Ethiopia is the number of informants selected to participate in the study. In this regard, many of the studies collected data from 17 to 38 informants [45, 54, 58, 65, 68] and concluded the knowledge is accumulated by the respected societies. Surprisingly, one study [45] collected ethnobotanical data from three traditional healers and concluded that the knowledge is acquired by local communities of the study area. These numbers could not be a representative sample size to elucidate the entire knowledge of indigenous people found in a district. Therefore, future ethnobotanical studies in Ethiopia should increase the number of informants to have a representative sample size. This will help to gather sufficient information about medicinal plant species. Many studies also did not compute ranking indices [23, 52, 56] though they have invaluable advantages in identifying the most preferred (important) medicinal plants in the study area which could aid phytochemical and pharmacological studies in searching of new drugs. Threats and conservation status of medicinal plants [21, 35, 54] were also overlooked by researchers, though they have advantages to using them sustainably. Ethnobotanical studies should also focus on identifying gender-based and age-based knowledge differences related to medicinal plant uses. In addition, future ethnobotanical studies in Ethiopia should focus on identifying most serious threats to medicinal plants and how local communities manage medicinal plants. Such studies will help in understanding the best conservation strategies and how local communities relate to the plant resources that they use as medicines.

The existing literature shows that there are several plant species having antidiarrhoeal activities. These plants have a lot of potential to be developed as alternatives to the existing drugs. Studies are needed to determine if, for the same active principle and at the same dosage, the efficiency is different for traditional and western remedies [72]. Despite the encouragement of promises shown by these materials, the applications have not been able to transfer from experimental animals' trials to humans and real-life applications; they all remained on papers. One of the drawbacks of the use of traditional medicinal plants for diarrhoeal diseases is the lack of precise dosage which may enhance toxicity [110]. In the assessment of the performance efficiencies of the different plant species as antidiarrhoeal properties, little efforts have been made to evaluate the toxicological effects of the extracts from these plant species. To ameliorate this challenge, attempts should be made to standardize (the drug preparation, dosage, and route of administration) and authenticate plant species which have antidiarrhoeal properties so that they could be used as natural drugs [111]. These efforts will help to produce effective drugs in treating various diarrhoeal diseases. Moreover, establishment of a medicinal plants database with important medicinal uses will have valuable advantages in searching of new drugs.

In searching of new drugs from medicinal plants overexploitation and the Nagoya protocol (2010) should not be overlooked. Overexploitation of medicinal plants especially those found in a restricted geographic location might lead to extinction. For example,* C. africana, Cucumis dipsaceus, S. kunthianum,* and* Withania somnifera* which were readily available in Ethiopia are now at the verge of extinction because of overexploitation [9, 54, 71]. Therefore, pharmaceutical companies and research institutions along with local communities should be encouraged to cultivate these medicinal plants to ensure the continuous supply of plant materials [112]. From a governance point of view, Ethiopia requires an enforceable policy that protects wild medicinal plants and policy incentives for the cultivation of medicinal plants to reduce overexploitation. Regarding the Nagoya protocol, fair and equitable sharing benefits arising from the utilization of genetic resources and community knowledge must be shared between the people using community knowledge and the people providing them as it is clearly stated in the agreement. If any product is used from the plant species for drug development obtained from indigenous knowledge, the local communities who acquired the knowledge should get benefit.

In order to obtain necessary information for optimum and practical conditions for antidiarrhoeal medicinal plants, there is also a need to investigate the physiological and biochemical functions demonstrated by these plant species. Additionally, identifying the individual bioactive natural products and elucidating the underlying mechanism of the process will have invaluable advantages to use the specific plant species for specific diarrhoeal diseases. Although different active components from various plant species are reported to have antidiarrhoeal activities, availability of similar amounts of those components among different plant species may not result in a similar antidiarrhoeal capabilities. The antidiarrhoeal activities within a single species may also vary depending on the type of climate, soil contents, crop year, and geographical features. Therefore, future phytochemical studies should consider the abovementioned conditions to produce the best out of plant species with antidiarrhoeal properties.

Phytochemical and pharmacological studies should have continued until the end users that experience the problem of diarrhoea. To do this, the dissemination of research findings to the appropriate governmental and nongovernmental agencies, who served as a boundary between the end users and the researchers, is necessary, but lacking. Thus, improvement on this channel of information dissemination shall be of great assistance in the promotion of the plant based antidiarrhoeal drugs to real life applications.

## 4. Conclusions

The study showed a rich diversity of indigenous medicinal plants commonly used for treatment of diarrhoeal diseases in Ethiopia. In this review paper, 132 medicinal plant species are reported that are used in the traditional diarrhoeal treatment by Ethiopian people. Ethnomedicinal research on distribution and usage pattern of antidiarrhoeal plants show variability across geographic and different regions in the country. For instance, sufficient studies were not conducted in the Afar, Benishangul Gumuz, Gambella, and Somali regions. Thus, researches should continue working in these areas that have received less attention. Use pattern of antidiarrhoeal herbal remedies and associated toxicity risks also need to be studied for future application without any doubt of observable side effects. Other challenges that need to be addressed are validation and efficacy of antidiarrhoeal plants. This could be addressed through in vivo and in vitro experiments with consideration to frequently used medicinal plants as to compete with existing conventional drugs. Any drug development needs clinical trials. This is the ultimate procedure to drug development for human or animal use.

## Figures and Tables

**Table 1:** Ethiopian medicinal plants used for treatment of diarrhoeal diseases. Description of data. Vernacular name (Or: Afaan Oromo; S: Somali; Ku: Kunama; T: Tigre, Am: Amharic; M: Maale; Me: Meinth; Sh: Shinasha; G: Gumuz; Si: Sidama) and parts used (L: leaf; B: bark; F: fruit; R: root; Bu: bulb; Fl: flower; S: seed; St: stem; Wp: whole plant). Cultivation (Cu: cultivated; Nc: not cultivated). An asterisk (*∗*) indicates data collected from traditional healers.

Scientific name	Vernacular name	Family	Parts used, preparation, and application	References
*Abutilon longicuspe* Hochst.	Polo (M)	Malvaceae	L	[20]^Nc^

*Acacia albida* Del.	Gerbi (Or, S)	Fabaceae	B, F, and L drunk orally (concoction) B crushed, homogenized in water, and drunk	[21]^Nc^ [22]

*Acacia tortilis *(Forssk.) Hayne	Tadacha, Dhadhacha (Or)	Fabaceae	Infusion L drunk orally	[21]^Nc^

*Acalypha villicaulis* A. Rich.	Zibute-kemun (Me)	Euphorbiaceae	R	[23]^Nc^

*Achyranthes aspera *L.	Maxxannee (Or)	Amaranthaceae	The L is crushed and mixed with water	[24]^Nc^

*Agave sisalana *Perrine	Angolaga (Ku) Eka (T)	Agavaceae	Chewing the internal (white) part of the R and swallowing the solution	[25]^Nc,*∗*^

*Ajuga alba *Robyns	Shai Shar (Aari)	Lamiaceae	L Crushed fresh L homogenized	[18, 20]^Nc^

*Ajuga integrifolia *Buch. Ham.	Zibute-kurijun (Me) Amaro (Am)	Lamiaceae	R Decoction of the fresh L is drunk	[23, 26]^Nc^

*Ajuga leucantha *Lukhoba	Shai sher (Aari)	Lamiaceae	L	[20]^Nc^

*Allium sativum *L.	Nech-shinkurt (Am)	Alliaceae	The bu are chewed and swallowed	[26]^Cu^

*Amaranthus caudatus *L.	Bahr tef (Am) Iyasu (Or)	Amaranthaceae	Squeeze the L and make a juice	[27–29]^Nc^

*Anogeissus leiocarpa* (DC.) Guill. & Perr.	Hanse (T) Sigaa (Am)	Combretaceae	B and L Fr are crushed in water and one cup is taken	[30]^Nc^ [31]

*Aristolochia bracteolata* RETZ.	Digel (Berta)	Aristolochiaceae	S grounded, dispersed in water, and drunk	[32]^Nc^

*Artemisia rehan *L.	Arity (Am)	Asteraceae	Boil L with water and drink the hot decoction	[27]^Cu^

*Asystasia guttata* Brummitt		Acanthaceae	R	[33]^Nc^

*Barleria argentea *Balf. fil.		Acanthaceae	R or L	[33]^Nc^

*Bersama abyssinica* Fresen.		Melianthaceae	Boil and drink the decoction (L)	[33]^Nc^

*Bidens pilosa* L.	Hachenti (M)	Asteraceae	R	[20]^Nc^

*Boscia angustifolia *A. Rich.	Vinna (Ku), Kermed (T)	Capparidaceae	Chewing	[25]^Nc,*∗*^

*Boswellia papyrifera* (Del.) Hochst.	Meker (Am)	Burseraceae	B	[30]^Nc^

*Brucea antidysenterica *J. F. Mill.	Wagnos (Am) Qomanyo (Or)	Simaroubaceae	Juice of crushed bark taken R powdered and mixed in water and drunk F powder mixed with honey	[34]^Cu^ [9] [35, 36]

*Cadaba rotundifolia *Forssk.		Capparaceae	Fresh L crushed and mixed with water to drink the concoction	[37]^Nc^

*Calpurnia aurea* (Ait.) Benth.	Ceekataa (Or) Digita (Sh, Am) Hitsawts (T) Kayneka/Digita (Aari) Zikita (Am)	Fabaceae	Fresh L crushed, boiled, and drunk Young shoot homogenized in water, decanted, and then drunk with honey The L of the young shoots from seven plants of Digita rubbed in the hands Fresh L soaked in water and taken orally S grinded, mixed with milk product locally called mancheba, and drink R F crushed, pounded, and drunk L is crushed, and the fluid is drunk Grind the S and eat after pounding with honey	[22, 23] [28]^Nc^ [38] [39] [40] [20] [41] [42] [43] [35]

*Capparis tomentosa* Lam.	Gaho (Sidama)	Capparaceae	L	[44]^Nc^

*Carica papaya* L.	Paapaayee (Or)	Caricaceae	S crushed and swallowed	[22]^Cu^

*Carissa spinarum *L.	Mitmita (Am)	Apocynaceae	Crush and squeeze the R	[31, 33]^Cu^

*Cissampelos mucronata* A. Rich.	Balari (M) Siyapewa (G)	Menispermaceae	R, crushed and squeezed	[20, 31]^Nc^

*Cissampelos pareira* L.	Abujelajil (G)	Menispermaceae	R, grounded and drunk with water	[32]^Nc^

*Citrus aurantifolia *Swingle		Rutaceae		[45]^Cu,*∗*^

*Citrus limon* (L.) Burm. fil.	Loomii (Or) Lendenan (S)	Rutaceae	Juice is drunk with pounded young L of *Cordia africana* The fruit is squeezed and the juice drunk	[28], [46]^*∗*^ [47]^Cu^

*Clematis hirsuta* Guill. & Perr.		Ranunculaceae	Crush the R, homogenize with cold water and drink	[33]^Nc^

*Clematis simensis *Fresen.	Hida Fiitii (Or)	Ranunculaceae	L are mixed in water and the solution is taken	[28]^Nc^

*Cleome gynandra *L.		Capparidaceae	Roasted S of the plant eaten	[18]^Nc^

*Clerodendrum myricoides* (Hochst) Vatke	Maraasisaa (Or) Misirich (Am)	Lamiaceae	R powder solution taken orally L	[48]^Nc^ [23]

*Clutia lanceolata *Forssk.	Fiyelefej (Am)	Euphorbiaceae	Crush the R and give with water	[23, 43]^Nc^

*Coffea arabica* L.	Buna (Or) Bunna (Am)	Rubiaceae	Roast the S, pounded and mixed with honey The roasted S is eaten or drunk in to empty stomach for 2-3 days Roasted powder taken with honey in empty stomach The dried coffee bean powdered and mixed with honey	[29]^Cu^ [49] [50] [24] [9, 26, 43, 51]

*Colocasia esculenta *(L.) Schott.	Goodarree (Or, G)	Araceae	Crush dry/fresh concocted with *Zingiber officinale* Rhizome is taken with coffee	[7]^Cu^, [52]^Cu,*∗*^

*Conyza bonariensis *(L.) Cronq.	Qilqilia (M)	Asteraceae	L	[23]^Nc^

*Conyza pyrrhopappa* A. Rich.	Donya (Dawaro)	Asteraceae	Crushed decocted L taken orally	[53]^Nc^

*Cordia africana* Lam.	Wanza (Am, Sh) Waddeessa (Or) Awhi (T) Banja (G)	Boraginaceae	Pound and squeeze the L by adding drop of water and drink the concoction by a cup of coffee. F (5–7) are eaten daily in the morning. B is crushed, homogenized in water, and drunk. Crush the L and drink it alone or by mixing it with boiled coffee R bark is crushed, squeezed with water, and given to drink	[37]^Cu^ [54] [55]^*∗*^ [28, 31] [40]

*Craterostigma plantagineum * Hochst.	Roba Enjire (Or)	Scrophulariaceae	R or L concoction taken orally	[56]^Nc^

*Croton macrostachyus* Hochst. ex Delile	Bakkanniisa (Or) Bissana (Am)	Euphorbiaceae	Dried L are powdered and mixed with hot water, and the mixture is taken orally L are eaten with *wat*	[55]^Cu,*∗*^ [35]^Nc^

*Cucumis dipsaceus* Ehrenb.	Hafaflo (T)	Cucurbitaceae	R	[30]

*Delphinium dasycaulon* Fresen.	Merbba (Ku)	Fabaceae	The R and L are pounded and drink a bottle cup of the juice in the morning	[25]^Nc,*∗*^

*Dodonaea angustifolia* L. f.		Sapindaceae	Boil the R and drink the decoction	[33]^Nc^

*Dorstenia barnimiana* Schwienf.	Work Bemeda (Am)	Moraceae	R powder mixed with honey and fermented for seven days. Then, taken orally in the morning until cured	[35]^Nc^

*Echinops cacrocheatus* L.	Keberich (Am)	Asteraceae	Pieces of St pounded, and the powder is taken	[57]^Cu^

*Eleusine coracana* (L.) Gaertn.	Dagusa (Am)	Poaceae	S	[58]^Cu^

*Embelia schimperi *Vatke		Myrsinaceae	Crush the F, homogenize with cold water and drink	[33]^Nc^

*Entada abyssinica *A. Rich.	Sesenaffa (ku)	Fabaceae	Pounding the R and drink a bottle cup of the juice in the morning	[25]^Nc,*∗*^

*Ensete ventricosum *(Welw.)	Wassa (Kocho)	Musaceae	Rhizome fermented, cooked and eaten	[59]^Cu^

*Erythrina brucei *Lam. Ex DC.		Fabaceae	Fresh inner B crushed and homogenized in water	[18]^Nc^

*Euphorbia candelabrum* Tremaut ex Kotschy	Karicho (Si)	Euphorbiaceae	L	[44]^Nc^

*Fagaropsis angolensis* (Engl.) Dale	Godicho (Si)	Rutaceae	L and Fl	[44]^Nc^

*Ficus sycomorus* Forssk.	Shola (Am) Fuka (G)	Moraceae	B juice is given orally R is crushed, soaked in water, and taken	[60]^Nc^ [31]

*Ficus thonningii* Blume.	Chibha (Am)	Moraceae	Chewing the R	[35]^Nc^

*Fuelugge virosa *(Willd.) Voigt.	Weela (G)	Euphorbiaceae	R is crushed and squeezed and one cup is taken	[31]^Nc^

*Gossypium barbadense *L.	Tite (Am)	Malvaceae	L powdered and mixed with water	[41]^Cu^

*Grewia villosa* Willd.	Bururi (K)	Malvaceae	Juice of L is taken orally.	[55]^Nc,*∗*^

*Guizotia abyssinica *(L.f.) Cass.	Nuugii (Or)	Asteraceae	Fried S, finely pounded and added as an ingredient to any concoction from medicinal plants	[28]^Cu^

*Heracleum abyssinicum *C. Norman	Ululee (Or)	Apiaceae	R is pounded together with R of *Thalictrum rhyncocarpum*	[28]^Nc^

*Hibiscus luduwigii* Eckl. and. Zeyh.		Malvaceae	R	[36]^Nc^

*Hoslundia opposita* Vahl	Dumdumach (Me)	Lamiaceae	R	[19]^Nc^

*Hypericum quartinianum* A. Rich.		Hypericaceae	L extracted with cold water	[61]^Nc^

*Hypoestes forsskaolii* (Vahl) R. Br.	Busino (M)	Acanthaceae	R	[20]^Nc^

*Indigofera spicata* Forssk.	Wesfat deshe (Aari)	Fabaceae	WP Crushed fresh R homogenized in water	[22]^Nc^ [18, 23, 36]

*Ipomoea obscura* (L.) Ker-Gawl.	Lago (M)	Convolvulaceae	L	[20]^Nc^

*Ipomoea purpurea *(L.) Roth		Convolvulaceae	Crushed fresh L homogenized in water	[18]^Cu^

*Kosteletzkya adoensis* (Hochst. ex A. Rich.) Mast.	Wari Beshe (M)	Malvaceae	L	[20]^Nc^

*Lagenaria siceraria* (Molina) Standl.	Busino (M)	Cucurbitaceae	L chopped and ground and drink the filtrate	[62]^Nc,*∗*^

*Lantana camara* L.	Yewef kollo (Am)	Verbenaceae	Dry St powder mixed with water and taken orally	[7]^Nc,*∗*^, [52]^Nc^

*Lantana ukambensis *Verdc.		Verbenaceae	Crushed fresh L homogenized in water	[18]^Nc^

*Leonotis ocymifolia* (Burm. F.) Iwarsson	Yeferes zeng (Am)	Lamiaceae	Dried L and Fr powder mixed with honey is given orally	[39]^Nc^

*Lepidium sativum *L.	Feecoo (Or) Shinfae (T) Fetto (Am)	Brassicaceae	S and Bu of *Allium sativum *are pounded Grind the S, mix with honey, and eat Mix the S powder in water and drink Grind the S and mix with honey and swallow	[63]^Cu^ [25]^*∗*^ [41] [30] [26]

*Leucas abyssinica *Briq.	Kirikisa (Dawaro)	Lamiaceae	Crushed and mixed with water and taken	[53]^Nc^

*Leucas deflexa* Hook. f.	Qechemen (B) Qilqilich (Me) Mezi (Sheko)	Lamiaceae	L	[23]^Nc^ [19] [64]

*Linum usitatissimum* L.	Telba (Am)	Linaceae	The water solution of roasted and powdered S is drunk in an empty stomach	[26]^Cu^

*Lippia javanica *Spreng.		Verbenaceae	R	[36]^Nc^

*Maesa lanceolata* Forssk.	Gowacho (Si)	Meliaceae	L and B	[33, 44]^Nc^

*Maytenus senegalensis *(Lam.)	Kbkib (T)	Celastraceae	Crush the L, mix it with milk, and drink	[40]^Nc^

*Melia azedarach* L.	Kiniinii (Or)	Meliaceae	B crushed, homogenized in water and drunk	[22]^Cu^

*Momordica charantia* L.		Cucurbitaceae	L and R ground well and mixed with milk and taken orally	[62]^Nc,*∗*^

*Momordica foetida* Schumach.	Yekurahareg (Am)	Cucurbitaceae	Pound the L, squeeze, and then drink	[36]^Nc^

*Musa paradisiaca* L.	Musi (Aari)	Musaceae	F	[20]^Cu^

*Myrtus communis* L.	Ades (Am)	Myrtaceae	Juice of L is taken orally in the morning	[35]^Nc^

*Nigella sativa* L.	Tikur azmud (Am)	Ranunculaceae	Leaves are pounded, and juice is prepared Concoct dry pounded seed	[57]^Cu^ [18]

*Nicotiana tabacum* L.	Tombowae (K)	Solanaceae	Crushed, decocted, and concocted fresh leaves are taken, and powdered roots are mixed with water or milk and drunk	[55]^Cu,*∗*^

*Ocimum forskolei* Benth.	Gurdarindo (M)	Lamiaceae	WP	[20]^Cu^

*Ocimum lamiifolium *Hochst. ex. Benth.	Damakese (Am)	Lamiaceae	Fresh L are pounded in water and the juice is drunk Crushed fresh L homogenized in water	[26]^Cu^ [25]^*∗*^

*Olinia rochetiana *A. Juss.		Oliniaceae	Boil the R and drink the decoction	[33]^Nc^

*Oryza sativa* L.	Ruz (Am)	Poaceae	One-half of a cup of coffee of squeezed L is drunk	[26]^Cu^

*Otostegia fruticosa *Sebald		Lamiaceae		[65]^Nc^

*Otostegia integrifolia* (Forssk.)		Lamiaceae	The L is crushed and squeezed then the concoction is drunk	[65]^Nc^

*Otostegia tomentosa* A. Rich.		Lamiaceae	The L is pounded and squeezed and drunk	[65]^Nc^

*Oxalis radicosa* A. Rich.	Solcarindo (M), Kinsa kins (Aaari)	Oxalidaceae	L	[20]^Nc^

*Pavonia urens* Cav.	Maxxannee (Or)	Malvaceae	R is chewed with salt and swallowed	[63]^Cu^

*Pentas lanceolata* (Forssk.) Deflers	Gaina deshe (Aari)	Rubiaceae	R L	[20]^Nc^ [23]

*Persea americana* Mill.	Abukato (Si)	Lauraceae	Dried F crushed, powdered, mixed with coffee and Drink	[59]^Cu^

*Phragmanlhera Macrosolen *(A. Rich) ined.	Diigaluu ceekaa (Or)	Loranthaceae	L is pounded and mixed with water	[63]^Nc^

*Plantago lanceolata *L.	Qorxobbii (Or)	Plantaginaceae	R is pounded with l of *Calpurnia aurea* and drunk with honey	[28]^Nc^

*Plectranthus cylindraceus *Benth.			Crushed and squeezed L is mixed in boiled cup of coffee and then the decoction is drunk	[65]^Nc^

*Plectranthus longipes* Baker	Solelo (M)	Lamiaceae	Wp	[20]^Nc^

*Plumbago zeylanica *L.	Aftuh (Am)	Plumbaginaceae	R	[30]^Nc^

*Podocarpus falcatus *C.N. Page	Birbirsa	Podocarpaceae	Chop the L, and drinking one coffee cup 2 times a day for three days	[66]^Nc^

*Pouzolzia parasitica* (Forssk.) Schweinf.	Dirba	Urticaceae	L and R crushed together and infusion is taken oral	[56]^Nc^

*Pseudocedrela kotschyi* (Schweinf.) Harms	Aduruba	Meliaceae	B eaten as it is	[32]^Nc^

*Pterolobium stellatum* (Forssk) Brenan	Qudu	Fabaceae	R boiled in water and drunk	[32]^Cu^

*Punica granatum *L.	Roomaan (Or)	Lythraceae	L boiled and drunk	[22]^Cu^

*Pycnostachys abyssinica* Fresen.	Anchip (M)	Lamiaceae	L	[20]^Nc^

*Rhus natalensis *Krauss	Kubri (M)	Anacardiaceae	L	[20]^Nc^

*Rubia cordifolia* L.	Anqis (Or)	Rubiaceae	Powder the R and drink the decoction	[61]^Nc^

*Rubus steudneri *Schweinf.	Gormach (Me)	Rosaceae	R Boil the R and drink the decoction	[19]^Nc^ [33]

*Rumex nepalensis *Spreng.	Germach (B) Tult (Am) Girshu (Sheko)	Polygonaceae	Crush R, mix with water, and drink Crush the R, mix with water, and then drink juice R and L	[23, 34]^Nc^ [27, 64] [43]

*Rumex nervosus* Vahl.	Dhangaggoo (Or) Embuacho (Am)	Polygonaceae	L crushed, homogenized in water, and drunk R powdered and mixed with melted butter	[23]^Nc^ [67]

*Salvia acuminata *Ruiz & Pav.	Anchino (M)	Lamiaceae	Chewing the L	[61]^Nc^

*Salvia nilotica* Juss. ex Jacq.	Shokoksaa (Or)	Lamiaceae	The f juice is taken orally	[55]^Nc,*∗*^

*Sapium ellipticum *(Hochst)		Euphorbiaceae	Decocted fresh L mixed with butter to drink	[18]^Nc^

*Schinus molle *L.	Tikur berbere (Am)	Anacardiaceae	Crush, the L filter and drink the fluid	[39, 68]^Cu^

*Senna didymobotry* (Freser) Irowin & Barinely	Sanamakii (Or)	Fabaceae	L are pounded, and juice is prepared	[57]^Nc^

*Senna obtusifolia* (L.) Irwin and Barneby		Fabaceae	S crushed, homogenized in water, and drunk	[22]^Nc^

*Senna singueana* (Delile) Lock	Karhaleko (M)	Fabaceae	R	[20]^Nc^

*Solanacio gigas *(Vatke) Jeffrey	Dilluu araba (Or)	Asteraceae	L and Fl are pounded together and taken	[28]^Nc^

*Solanum giganteum *Jacq.		Solanaceae	R	[36]^Nc^

*Solanum nigum *L.	Awut (Am)	Solanaceae	Crush the L, chew, and then swallow the juice	[43]^Nc^

*Sorghum bicolor* (L.) moench	Bisingaa (Or) Zengada (Am)	Poaceae	The S is powdered and made into a porridge S	[69]^Cu^ [58]

*Sporobolus pyramidalis *Beauv.	Gic'igiliya (Dawro)	Poaceae	L powdered and mixed with the L of *Conyza pyrrhopappa *and taken	[53]^Nc^

*Stereospermum kunthianum* Cham.	Zana (Ag), Wahsinte (Am) Estegne eyo (Berta)	Bignoniaceae	R grounded, dispersed in water, and drunk R is crushed, diluted in water, and one cup is taken	[54]^Nc^ [31]

*Stylosanthes fruticosa* (Retz.) Alston	Dino desho (M)	Fabaceae	L	[20]^Nc^

*Syzygium guineenses *(Willd.) DC.	Dokima (Am) Diwa (G)	Myrtaceae	Decoction of B powder St bark is crushed, squeezed and taken orally	[69]^Nc^ [31, 43]

*Tagetes minuta *L.	Kawato (M) Hada Gola (Am)	Asteraceae	L decocted	[62]^Nc,*∗*^ [70]

*Tamarindus indica *L.	Koriya (Dawro)	Fabaceae	F crushed and mixed with water and taken orally	[53]^Cu^

*Terminalia brownii* Fresen.	Weyba (T) Rukessa (Si)	Combretaceae	B and L L	[30] [44]^Nc^

*Termnalia schimperiana* Hochst	Biguha (G)	Combretaceae	B is crushed, diluted in water, and taken orally	[31]^Nc^

*Thalictrum rhynchocarpon *Quart.		Ranunculaceae	Boil the R and drink the decoction Crushed fresh L homogenized in water	[33]^Nc^ [18]

*Tragia mitis *ex A. Rich.		Euphorbiaceae	Crushed R, mixed with water and sugar	[70]^Nc^

*Trigonella foenum-graecum *L.	Abeake (T)	Boraginaceae	Add the s into boiled water which contain *Zingiber officinale,* allow it to boil for a while, and then drink a cup of the decoction	[25]^Nc,*∗*^

*Verbascum sinaiticum* Benth.	Gurraharree (Or) Kutitina (Am) Daba Keded (Am)	Scrophulariaceae	L crushed, homogenized in water, and drunk Crush the R and drink with water Juice of R is taken orally	[22]^Nc^ [43] [35] [36]

*Verbena officinalis* L.	Atuch (Am) Q/albaatii (Or)	Verbenaceae	R powder mix with water Decant from pounded root is drunk The crushed R is left in water for some time and then the extract is drunk	[54]^Nc^ [67] [28] [26] [24]

*Vernonia amygdalina *Del.	Eebicha (Or, G)	Asteraceae	Crush the L and mix with little water and then drink for five days Crush the L, pound and mix with little water and then drink for five days	[52]^Cu,*∗*^ [69] [7]^*∗*^ [33]

*Vernonia auriculifera *Hiem	Aegidim (G)	Asteraceae	R is crushed and squeezed and one cup is taken	[31]^Nc^

*Viscum tuberculatum *A. Rich	Dhigri (S)	Viscaceae	R grounded, boiled with water, and drunk	[47]^Nc^

*Withania somnifera* (L.) Dunal.	Gizawa (Am)	Solanaceae	Infusion powder of 3 L mixed with water	[71]^Nc^

*Ximenia americana* L.	Feya (Gumuz)	Olacaceae	Crushing and squeezing the R	[31]^Nc^

*Zehneria scabra *(Linn. f.) Sond.	Hidda bofaa (Or) Hidda reffaa (Or) Hareg resa (Am) Areg resa (Am)	Cucurbitaceae	L are pounded, and juice is prepared The juice of fresh squeezed L is drunk Crush the L and drink The juice of fresh squeezed leaves is drunk	[57]^Nc^ [69] [43] [26] [33]

*Zingiber officinale *Roscoe.	Zingibil (T, Am)	Zingiberaceae	Chew and swallow the fluid	[40]^Cu^ [33]

*Zinnia peruviana* L		Asteraceae	R	[36]^Nc^

*Ziziphus mauritiana *Lam.	Amurusam (Berta)	Rhamnaceae	S is ground, dispersed in water, and drunk	[32]^Nc^

*Ziziphus spinachristi* (L.) Desf.	Kurkura (Or); Gob, Geb (S) Sirra	Rhamnaceae	The concoction of L drunk orally St bark is crushed, soaked in water, and filtered	[21]^Nc^ [31]

**Table 2:** Taxonomic diversity of medicinal plants in the study area.

Family	Number of genera	Percentage	Number of species	Percentage of species
Fabaceae	10	9.0	12	9.1
Lamiaceae	7	6.2	12	9.1
Asteraceae	8	7.1	10	7.5
Euphorbiaceae	7	6.2	7	5.3
Cucurbitaceae	3	2.6	4	3.0
Malvaceae	4	3.5	4	3.0
Poaceae	4	3.5	4	3.0
Ranunculaceae	3	2.6	4	3.0
Solanaceae	3	2.6	4	3.0
Verbenaceae	3	2.6	4	3.0
Rubiaceae	3	2.6	3	2.3
Rutaceae	2	1.7	3	2.3
Moraceae	2	1.7	3	2.3
Other 39 families	54	47.7	58	44.0
Total	113	100.0	132	100.0

**Table 3:** Method of preparation of traditional medicine in Ethiopia.

Method of preparation	Number of preparations	Percentage
Crushing	53	38.2
Decoction	30	21.5
Powdered	14	10.1
Chewing	7	5.0
Concoction	5	3.6
Others	30	21.5
